# Comparison between hyperechoic and normo-echoic amniotic membranes in patients with preterm premature rupture of membranes regarding pregnancy outcome

**DOI:** 10.1007/s00404-025-08051-1

**Published:** 2025-05-20

**Authors:** Tamer Yehia M. Ali, Bassant F. Elsayed, Ahmed A. Aboelroose, Mohamed M. Farrag, Ahmed M. Gadallah

**Affiliations:** Department of Obstetrics and Gynecology, Faculty of Medicine, Suez Canal University, Ismailia, Egypt

**Keywords:** Preterm, Rupture membranes, Amniotic membranes, Pregnancy outcome

## Abstract

**Purpose:**

To evaluate the amniotic membrane echogenicity as a marker of early delivery among pregnant women presenting with leakage of amniotic fluid.

**Methods:**

This prospective cohort study was commenced at the Obstetrics and Gynecology Department at Suez Canal University Hospital from March 2023 to March 2024. The study recruited 72 pregnant females aged 20–45 years with singleton pregnancies and presented with preterm leakage of amniotic fluid. The gestational age was between 28 and 37 weeks. The sonographic appearance of the amniotic membranes close to the internal os was evaluated using transvaginal ultrasound. The membranes were classified as hyperechoic when they showed echogenic similarity to the fetal bones (either skull, femur, or pelvic bones) or normo-echoic. The primary outcome measures were the time interval from admission to delivery and the incidence of spontaneous preterm labor.

**Results:**

The mean age of patients was 26.06 years old, with mean gestational age at hospital entry 32.47, with no statistically significant difference between hyperechoic and normo-echoic membranes groups. The admission to delivery time was longer among the normo-echoic group (20 days versus 7.1 days,* p* value = 0.001). The incidence of spontaneous preterm delivery was 71.9% in the hyperechoic membranes group versus 50% in the normo-echoic membrane group.

**Conclusion:**

The existence of fetal membranes with increased echogenicity overriding the cervix could anticipate an unavoidable preterm birth in patients diagnosed with preterm premature rupture of membranes.

## What does this study add to the clinical work

**Table:** 

Few studies evaluated membrane echogenicity among women with premature membrane rupture.The current study reported a strong association between increased membrane echogenicity and decreased total latency period duration.	

## Introduction

Fetal membranes secure a protective environment for the fetus in the uterus, and its rupture occurs during the uterine contraction at delivery [1, 2]. Suppose the fetal membranes rupture during pregnancy before 37 weeks of gestation. In that case, this is known as preterm premature rupture of membranes (P-PROM) [2], which accounts for about one-third of all recorded preterm deliveries [3], thus leading to multiple neonatal and maternal morbidities and mortalities [4].

Until now, there has been no exact resolution of the direct etiological mechanisms behind the incidence of P-PROM, and the condition is multifactorial and has multiple risk factors [1, 5]. Vaginal colonization is the most common factor associated with P-PROM. Women can be divided into group B streptococcus (GBS) colonized and non-GBS colonized since its effect on the newborn is paramount. However, till now, there is no solid evidence to link between P-PROM and maternal colonization with GBS [6].

Multiple studies have previously examined different methods for detecting P-PROM and near delivery. However, they were either poor predictors, such as the cervical length at transvaginal ultrasound, or not feasible and could not be widely used. However, they showed promising results for intra-amniotic assessment of inflammatory markers [7, 8].

Identifying an easy-to-use, significant predictor of women with P-PROM who are at impending delivery would help significantly improve the neonatal and maternal outcome via early referral to specialized centers with neonatal intensive care units (NICU) and early initiation of strict clinical monitoring and steroids with or without magnesium sulfate administration [9, 10].

The current study evaluated the amniotic membranes'echogenicity as a marker for spontaneous preterm labor among pregnant women complaining of P-PROM.

## Methods

This prospective cohort study was conducted at the obstetrics and gynecology department at Suez Canal University Hospital from 15/4/2023 till 15/4/2024. The study recruited women with P-PROM following inclusion and exclusion criteria. Inclusion criteria: (a) 20–45 years, (b) single baby, (c) gestational age between 28 and 37 weeks, and (d) nulliparous or multiparous women. Exclusion criteria: (a) abnormalities of placentation, (b) previously diagnosed uterine malformation, (c) cervical cerclage, (d) congenital infections, (e) fetal abnormalities, (f) presented with uterine contractions, and (g) evidence of chorioamnionitis.

We recruited women with P-PROM throughout the study period. Patients were diagnosed with P-PROM based on the clinical history and speculum examination (presence of amniotic fluid leakage from the cervical os during sterile speculum examination) [11]. Patients were followed up from diagnosis until spontaneous onset of labor.

Eligible patients were subjected to:

- Laboratory tests include cervical and vaginal swabs, mid-stream urine culture, complete blood count, and C-reactive protein (CRP).
- A transabdominal ultrasound assessed fetal viability, presentation, estimated fetal weight, and amniotic fluid index (AFI).
- Transvaginal ultrasound measured cervical length along its longitudinal axis with an empty bladder from the internal to the external os [12]. The sonographic appearance of the amniotic membranes close to the internal os was evaluated. The membranes were classified as hyperechoic when they had an echogenic appearance similar to the fetal bones (either skull, femur, or pelvic bones) or normal [13].
- Maternal follow-up based on the daily monitoring of clinical signs of chorioamnionitis (diagnosed by elevated body temperature ≥ 38 degrees together with maternal tachycardia, fetal tachycardia, uterine tenderness, foul odor, vaginal discharges, and an increased total leucocytic count) [14]. Laboratory tests (white blood cells and C-reactive protein) were recorded on alternate days. Fetal follow-up and surveillance were carried out using daily cardiotocography (CTG) and biweekly transabdominal ultrasound with umbilical Doppler.
- Active labor was defined by a fully effaced, ≥ 6 cm dilatated cervix coupled with > 3 contractions in 10 min recorded at tocography [15]. Delivery was expedited in cases of possible chorioamnionitis or fetal compromise. In contrast, in clinically stable cases, a policy of expectant management was adopted until 37 + 0 weeks of gestation, except in those cases with a documented Group B streptococcus infection, in whom delivery was offered at gestations > 34 weeks [16]. Spontaneous preterm labor was not inhibited by tocolytic. In case of delivery < 32 + 0 weeks, magnesium sulfate for improved neural function of the fetus was administrated at least two hours before delivery. Based on the obstetric indications, elective delivery was carried out using induction of labor (IOL) or cesarean section (CS).

The primary outcome measure was to contrast the admission-to-delivery interval between patients of P-PROM with and without hyperechoic membranes. The other outcome measure was the rate of spontaneous preterm labor.

### Statistical analysis

The gathered information was processed using SPSS version 23 (SPSS Inc., Chicago, IL, USA.). Quantitative data were expressed as means ± SD, while the qualitative data were expressed as numbers and percentages (%). The normality of data was tested using the Shapiro–Wilk test, and statistical tests were used accordingly. Comparison between groups was tested using an independent Student *t* test for continuous variables and a Chi-square test for categorical variables. A probability value (*p* value) < 0.05 was considered statistically significant. Binary logistic regression was done on a 2-step approach. The first step was the univariate regression, where each variable was tested against the outcome to determine the crude odds ratio (OR). Significant variables were put into a multivariate regression model, producing a model with more than one variable and giving the adjusted OR for each variable. Data were presented as relative risk and a confidence interval of 95%.

## Results

**Fig. 1:**
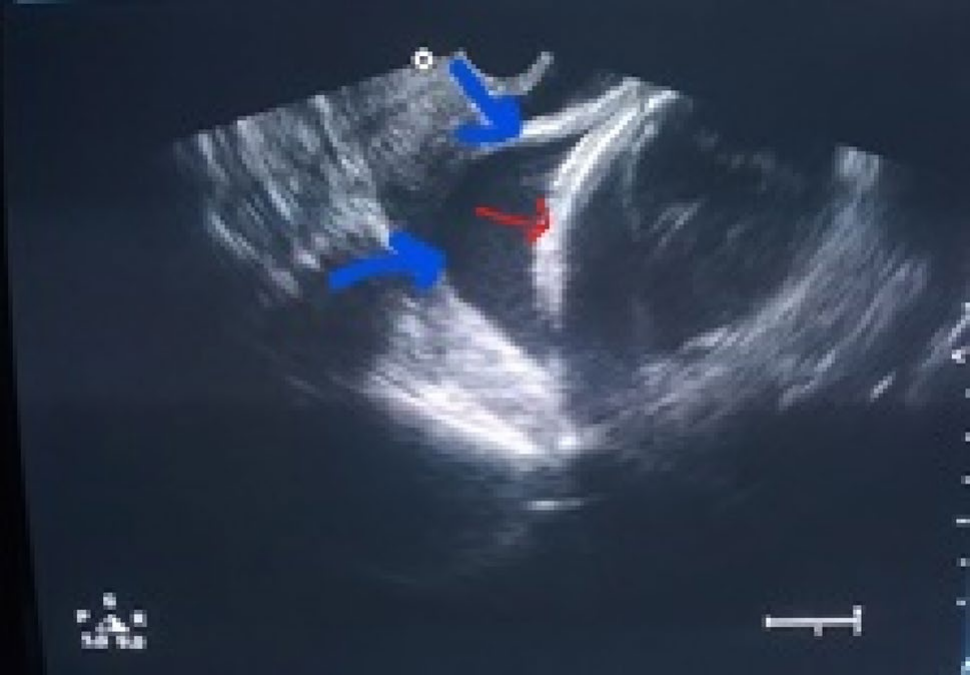
Appearance of normo-echoic membrane. The blue arrows demonstrate the fetal membranes that differ in echogenicity from the fetal skull (red arrow)

**Fig. 2:**
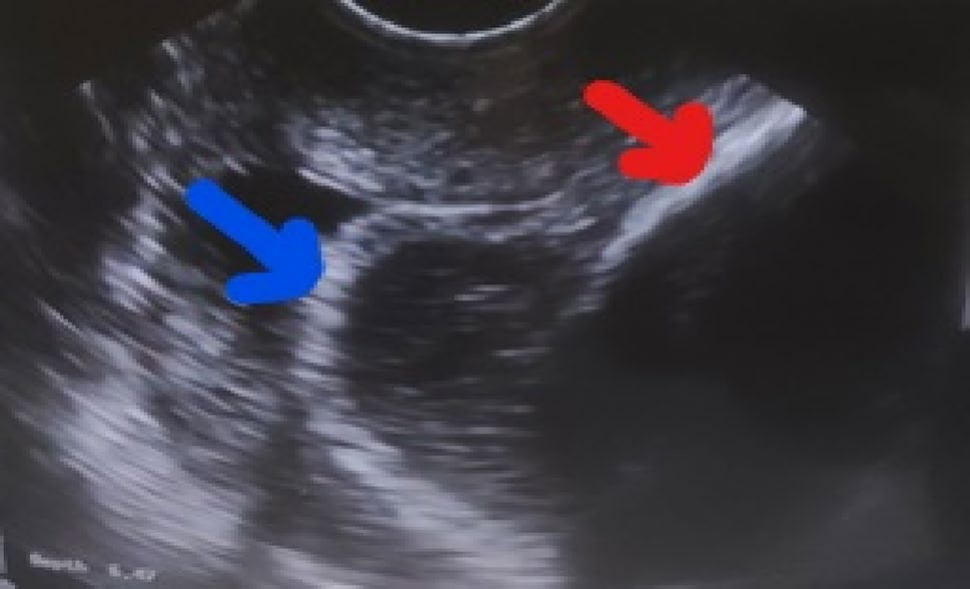
Appearance of hyperechoic membrane: The blue arrow demonstrates fetal membranes of the same echogenicity as the fetal skull (red arrow)

**Fig. 3:**
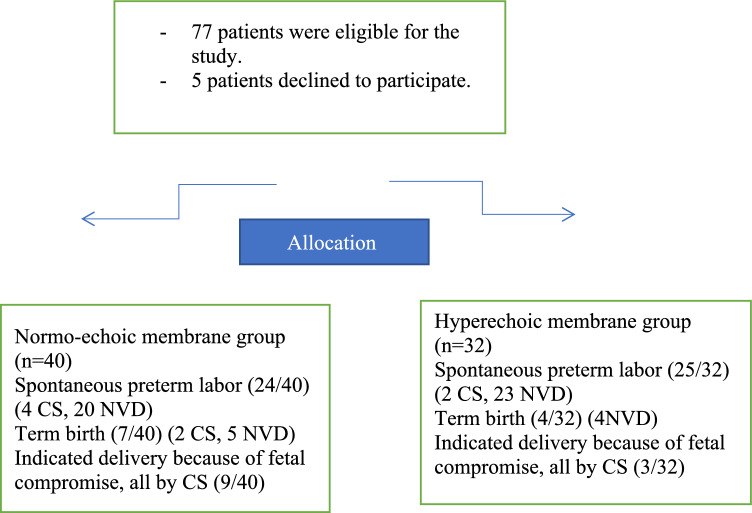
Patients’ flow chart

Seventy-seven patients were eligible for the study. Five women declined to participate. Accordingly, a total of 72 pregnant females presented with preterm premature rupture of membranes were recruited. Based on the transvaginal ultrasound findings, 40 had normal echoic maternal membranes (Fig. 1), and 32 had hyperechoic membranes (Fig. 2). In the normo-echoic membrane group, 24/40 women had a spontaneous preterm birth, 7/40 women had term delivery (2 women required CS, while five women delivered vaginally), and 9/40 women had indicated CS because of fetal compromise. In the hyperechoic membrane group, 25/32 women had spontaneous preterm birth, 4/32 women had term vaginal delivery, and 3/32 women had indicated CS because of fetal compromise (Fig. 3).

Both groups were matched regarding maternal age and gravidity, with a mean age of 26.06. Most of the studied females in both groups were multigravida. At admission, the mean gestational age was 32.47 weeks and was found to be significantly higher among females with membranes of increased echogenicity. The estimated fetal weight was significantly higher among females with hyperechoic membranes. The cervical length showed no significant difference between both groups (Table 1).

**Table 1:** patients'characteristics

Variable	Total	Echogenicity of membranes		*p* value
		Hyperechoic (*n* = 32)	Normoechoic (*n* = 40)	
Maternal age (years) (mean ± SD)	26.06 ± 4.9	27.06 ± 5.58	25.25 ± 4.33	0.1 (NS)
Gravidity				0.6 (NS)
Primi-gravida	27 (37.5%)	13 (40.6%)	14 (35%)	
Multigravida	45 (62.5%)	19 (59.4%)	26 (65%)	
Gestational age at admission (weeks) (mean ± SD)	32.47 ± 2.45	33.3 ± 2	31.8 ± 2.6	0.01*
Cervical length (mm) (mean ± SD)	32 ± 1.7	31.8 ± 1.6	32.12 ± 1.77	0.7 (NS)
EFW (Kg) (mean ± SD)	1.9 ± 0.49	2.08 ± 0.39	1.7 ± 0.5	0.002*
TLC (×10^3^µl) (mean ± SD)	9.84 ± 2.33	9.78 ± 2.5	9.89 ± 2.22	0.610
CRP (mg/dl) (mean ± SD)	3.4 ± 0.9	3.26 ± 0.91	3.51 ± 0.89	0.108

*Statistically significant difference, *EFW* estimated fetal weight, *TLC* total leucocytic count, *CRP* C-reactive protein, *NS* no statistically significant difference

The gestational age at delivery showed no statistically significant difference according to membrane echogenicity. However, the time interval from admission to delivery was significantly longer among females with normo-echoic membranes, 20 days, versus 7.1 days among females with hyperechoic membranes. Spontaneous preterm delivery was reported among 71.9% of females with membranes of increased echogenicity versus 50% among females with normo-echoic membranes, with a statistically significant difference (Table 2).

**Table 2:** Pregnancy outcome according to membrane echogenicity

Variable		Total	Echogenicity of membranes		*p* value
			Hyperechoic (*n* = 32)	Normoechoic (*n* = 40)	
Gestational age at delivery (weeks)	Mean ± SD	34.2 ± 2.2	34.01 ± 2.17	34.36 ± 2.24	0.5 (NS)
Admission to delivery interval (days)	Mean ± SD	14.3 ± 11.8	7.1 ± 5.6	20 ± 12.3	0.001*
Spontaneous PTL		49 (68.1%)	25 (78.1%)	24 (60%)	0.2 (NS)

*NS* No statistically significant difference

*Statistically significant difference

The risk of spontaneous preterm delivery among females presented with preterm premature rupture of membranes was found to be 1.44 times among those with membranes of increased echogenicity when compared with those with normo-echoic membranes with 95% confidence interval 0.9–2.09 but without statistical significance (Table 3).

**Table 3:** Risk of spontaneous preterm labor according to membrane echogenicity

Membrane echogenicity	Spontaneous PTL		*p* value	Relative risk (95% CI)
	No	Yes		
Normoechoic	16 (40%)	24 (60%)	0.1 (NS)	1.44 (0.9–2.09
Hyperechoic	7 (21.9%)	25 (78.1%)		

*NS* No statistical significance

When comparing women who had spontaneous onset of labor and those who had indicated delivery revealed a significant difference in the gestational age at admission and delivery. Women with a spontaneous onset of labor were more preterm upon admission and at delivery (*p* values 0.001 and 0.014, respectively). Fetal weight was significantly lower among those with a spontaneous onset of labor (1805.5 ± 480.8 vs 2249.9 ± 394.7, *p* value 0.002). Most cases with spontaneous onset of labor delivered preterm 49/58 (84.5%) rather than term 9/58 (15.5%). The indicated delivery was mainly due to fetal compromise 12/14 (85.7%) (*p* value 0.0001). Spontaneous onset of labor occurred in nulliparous women in a significant proportion, 34/58 (58.6%), while indicated delivery was evident among multiparous women 11/14 (78.6%) (*p* value 0.012). Neither group had a significant difference in membrane echogenicity (Table 4).

**Table 4:** Comparison between study participants according to the onset of labor

	Spontaneous onset (58/72)	Indicated delivery (14/72)	*p* value
Age (years) (mean ± SD)	26.02 ± 5.3	26.21 ± 3.4	0.895
GA at admission (weeks) (mean ± SD)	32.02 ± 2.4	34.4 ± 1.5	0.001
GA at delivery (weeks) (mean ± SD)	33.9 ± 2.3	35.5 ± 0.9	0.014
Latency period (days) (mean ± SD)	13.24 ± 12.1	7.9 ± 10.2	0.132
CL (mm) (mean ± SD)	31.9 ± 1.7	32.2 ± 1.8	0.603
EFW (gm) (mean ± SD)	1805.5 ± 480.8	2249.9 ± 394.7	0.002
TLC (×10^3^µl) (mean ± SD)	9.8 ± 2.4	9.9 ± 2.1	0.868
CRP (mg/dl) (mean ± SD)	3.4 ± 0.9	3.6 ± 0.9	0.474
Membrane echogenicity			0.053
Normo-echoic	29 (50%)	11 (78.6%)	
Hyperechoic	29 (50%)	3 (21.4%)	
Time of delivery			0.0001
Preterm birth	49 (84.5%)	0 (0%)	
CS for fetal compromise	0 (0%)	12 (85.7%)	
Term	9 (15.5%)	2 (14.3%)	
Parity			0.012
Nullipara	34 (58.6%)	3 (21.4%)	
Multipara	24 (41.4%)	11 (78.6%)	

*GA* gestational age, *CL* cervical length, *EFW* estimated fetal weight, *TLC* total leucocytic count, *CRP* C-reactive protein

Binary logistic regression for the factors associated with spontaneous birth before and after 72 h from the onset of membrane rupture was performed. Normoechoic membranes were associated with significantly prolonged gestation and delivery after 72 h (*p* value < 0.001). A longer cervical length was associated with prolonged gestation (*p* value 0.007). In addition, increased body temperature was associated with delivery before 72 h of membrane rupture (*p* value 0.004) (Table 5).

**Table 5:** Binary outcome logistic regression analysis to detect significant independent predictors of spontaneous labor among studied patients before and after 72 h from the onset of membrane rupture

Group	≤ 72 h	> 72 h	*p* value	Crude OR (95% CI)	Adjusted OR (95% CI)
Normoechogenic, n (%)	7 (26.9)	33 (71.7)	< *0.001*	0.145 (0.049–0.427)	
Age	27 [21–31]	24 [30–22]	0.837		
Nulliparous, n (%)	14 (53.8)	23 (50)	0.754		
GA on admission	33.8 [32.1–35]	32.5 [34.3–29.3]	0.061		
GA at delivery	34.1 [32.3–35.3]	35 [36.6–33]	*0.026*	1.288 (1.023–1.620)	9.471 (3.025–29.655)
CL	31 [30–32]	32 [34–31]	*0.007*	1.580 (1.123–2.223)	
EFW	2051 [1800–2400]	1837 [2200–1350]	*0.03*	0.999 (0.998–1.000)	0.989 (0.984–0.995)
WBC, n × 10⁹/L	9.6 [9–12]	9.9 [11.1–7.8]	0.189		
Temperature	37 [36.7–37.1]	36.5 [37–36]	*0.004*	0.232 (0.080–0.671)	
CRP, mg/dl	3.4 [3–4]	3.5 [4.1–2.7]	0.991		

*WBC* white blood cells, *CRP* C-reactive protein, *EFW* estimated fetal weight. Expression of quantitative variables in median [interquartile range adjusted OR calculated for significant variables on univariate analysis]

## Discussion

The most common site for membrane rupture is the membrane overlying the cervical os. It is structurally different, disrupted easily, and is commonly loaded with bacteria [17]. However, not all cases of PROM occur according to this theory [18].

Hyperechoic membranes were reported in 32/72 cases (44.4%) with P-PROM. Membranes with increased echogenicity were attributed to an inflammatory process among women with echogenic membranes. This inflammatory process leads to specific biochemical and histological changes that raise the echogenicity of the membrane overlying the cervix [19]. Another explanation would be the existence of edema and exudation of the collagen network within the different layers of the membrane [20, 21], loss of water, and the accumulation of inflammatory proteins and collagen degradation [13].

The overall mean latency period of the studied population was 14.3 ± 11.8. An earlier study reported a median latency period of 6 days [22]. Others reported a window of 4–13 days for the latency period [23]. Membranes with increased echogenicity were linked to a significantly shorter admission to delivery interval and increased possibility of spontaneous preterm delivery. These findings agreed with Volpe and colleagues, who mentioned that increased membrane echogenicity in women with P-PROM was linked to a significantly shorter latency period [13]. Another research mentioned that advanced gestational age at admission was linked to a shorter latency period [22], evident in the hyperechoic membrane group. This represents a reflection of the advanced inflammatory process that eventually leads to preterm labor and delivery [13].

An earlier study reported a sixfold increased risk for spontaneous preterm birth among women with hyperechoic membranes [13], while the current study reported a 1.44 increased risk, which was not statistically significant.

Membrane echogenicity, cervical length, and increased body temperature affected the onset of labor among women with premature membrane rupture. Normoechoic membranes and increased cervical length were significantly associated with prolonged gestation > 72 h. An earlier study reported that hyperechoic membranes were significantly associated with an onset of labor < 72 h, while increased cervical length was associated with prolonged gestation [13], which was following the current study. This was rendered to an early onset of inflammation affecting the membranes upon the cervix, resulting in increased echogenicity and early labor onset [19]. Another study reported nulliparity, advanced gestational age at admission, and oligohydramnios were associated with a short latency [22]. This discrepancy was attributed to different etiological factors for PROM among the studied populations [24].

### Strength and limitations

The study was conducted as a prospective cohort study. The same radiologist did the ultrasound to avoid possible interobserver variability. The main limitation of the current study is the small sample size. Membrane echogenicity was evaluated subjectively, which might affect the accuracy of the evaluation. Although the reported results were from previous studies, further studies are still required, and extending follow-up to detect neonatal outcomes is essential. It was conducted as a single-center study, which limits the generalizability of the results.

### Research implications

Further studies evaluating the association between membrane echogenicity and histological evidence of infection would be more informative. Additionally, the association between membrane echogenicity and neonatal outcomes is required.

## Conclusion

Membrane echogenicity is a significant independent risk factor for spontaneous preterm delivery and is also significantly associated with a shorter admission-to-delivery time interval. Identifying patients at higher risk of early onset spontaneous preterm delivery will guide us to early referral to specialized centers with NICUs and early initiation of therapy.

## Data Availability

Data are available upon reasonable request from the corresponding author.
